# Assessing Nutritional Knowledge and Physical Health Among Football Players: A Pilot Study from Three Sports Clubs in Western Romania

**DOI:** 10.3390/sports13010016

**Published:** 2025-01-09

**Authors:** Gabriel Roberto Marconi, Brigitte Osser, Gyongyi Osser, Caius Calin Miuța, Csongor Toth, Viorel Petru Ardelean, Anca Dicu, Corina Dalia Toderescu, Laura Ioana Bondar

**Affiliations:** 1 Faculty of Physical Education and Sport, “Aurel Vlaicu” University of Arad, 310130 Arad, Romania; gabriel.marconi@uav.ro (G.R.M.); gyongyi.osser@uav.ro (G.O.); csongor.toth@uav.ro (C.T.); viorel.ardelean@uav.ro (V.P.A.); 2 School of Biomedical Sciences, University of Oradea, 410087 Oradea, Romania; bondar.lauraioana@student.uoradea.ro; 3 Faculty of Food Engineering, Tourism and Environmental Protection, “Aurel Vlaicu” University of Arad, 310130 Arad, Romania; anca.dicu@uav.ro; 4 Faculty of Medicine, “Victor Babeș” University of Medicine and Pharmacy, 300041 Timișoara, Romania; corina.toderescu@umft.ro; 5 Department of Biology and Life Sciences, “Vasile Goldiș” Western University of Arad, 310048 Arad, Romania

**Keywords:** athlete wellness, dietary habits, football players, health behaviors, nutritional knowledge, physical health, sports nutrition, substance use, training frequency

## Abstract

Background: Football players require optimal nutrition and physical fitness to enhance their performance and maintain their health. Understanding the relationships among nutritional knowledge, dietary habits, physical health, and substance use in athletes is essential for developing effective strategies. This study investigates these factors in male football players aged 16–33 years. Methods: The study involved 60 male football players from three teams in Liga4Arad. A mixed-methods approach was used, incorporating a self-developed pilot questionnaire and internationally validated instruments. The reliability of the questionnaire was confirmed using Guttman’s λ2. The questionnaire assessed nutritional knowledge, eating habits, substance use, and physical health parameters, including body fat percentage and training frequency. Spearman’s correlation was used to analyze the data and explore the interrelationships between these factors. It is important to note that the pilot questionnaire used in this study was self-developed and not previously validated in this specific context. Results: Nutritional knowledge was positively correlated with healthier eating habits (*ρ* = 0.675, *p* < 0.001). Intensive training and higher physical activity levels were both associated with improved body composition and lower body fat (*ρ* = 0.341, *p* = 0.006). Supplement use was moderately correlated with alcohol consumption (*ρ* = 0.548, *p* < 0.001) and weakly correlated with smoking (*ρ* = 0.348, *p* = 0.007). Conclusions: The study highlights a strong relationship between nutritional knowledge and healthier eating habits among football players, as well as the significant role of frequent intense training in reducing body fat percentages and enhancing physical fitness. Additionally, the findings suggest a moderate association between dietary supplement use and alcohol consumption, underscoring the need for tailored interventions to address substance use and its impact on players’ health behaviors and performance.

## 1. Introduction

Football, one of the most popular and physically demanding sports worldwide, requires players to maintain optimal health, fitness, and performance levels [1,2]. For football players, a balanced diet and an appropriate training regimen are critical in achieving peak physical condition [3,4]. In addition to the physical aspect, the knowledge and application of proper nutrition can significantly influence an athlete’s performance, recovery, and overall health [5,6]. Given the competitive nature of the sport, understanding the relationship between nutritional knowledge, dietary habits, substance use, and physical fitness is crucial for optimizing training and performance outcomes [7,8].

In recent years, research has increasingly focused on the importance of nutrition in sports performance, particularly among professional athletes [9,10]. Studies have shown that athletes with higher nutritional knowledge are more likely to engage in healthier eating behaviors, which contribute to enhanced performance and reduced injury risk [11,12]. Conversely, poor dietary choices can have detrimental effects on body composition, recovery times, and overall physical health [13,14]. Alongside nutrition, substance use, including dietary supplements, alcohol, and smoking, has been a subject of concern, with some research suggesting that certain substances may negatively impact athletes’ health and performance [15,16,17,18].

Despite these findings, the relationship between nutritional knowledge, eating habits, substance use, and physical health remains a complex and underexplored area in sports science. Some studies suggest a direct link between higher nutritional knowledge and improved dietary practices, while others highlight the influence of external factors such as social pressures, peer behavior, and mental stress [19,20,21,22]. Additionally, the impact of substance use on athletic performance and health behaviors is still debated, with some research indicating a potential negative correlation between supplement use and other health risk behaviors, while others suggest that these factors may be unrelated or influenced by different psychological factors [23,24,25].

The purpose of this study is to explore the interrelationships between nutritional knowledge, dietary habits, physical health parameters, and substance use behaviors (specifically, the consumption of dietary supplements, alcohol, and tobacco) among male football players aged 16–33 years. By investigating these factors, the study aims to gain a deeper understanding of how these variables influence the overall health and performance of athletes. The significance of this research lies in its potential to inform sports nutrition strategies, help improve training regimens, and promote healthier lifestyles for athletes, particularly in football.

The rationale for this study stems from the need to address the gaps in the current literature regarding the relationship between dietary knowledge, habits, and substance use among football players. While previous studies have examined various aspects of nutrition and health behaviors in athletes, limited research has specifically targeted football players, particularly in the context of their physical health and performance [26,27]. Furthermore, there is a need for more robust data on how different factors—such as substance use and training frequency—interact to influence an athlete’s overall well-being. By addressing these issues, this study aims to contribute valuable insights to the field of sports nutrition and athlete health, ultimately offering practical recommendations for improving player performance and promoting a balanced, healthy lifestyle in football.

## 2. Materials and Methods

### 2.1. Study Design and Setting

This cross-sectional study was conducted between October 2023 and October 2024 in Arad, Romania. The research focused on football players from three local clubs: Vointa Macea, Soimii Simand, and Athletico Vinga. Data collection was carried out at the clubs’ training facilities to ensure convenience and minimize disruption to the players’ training schedules.

### 2.2. Study Population

The study involved 60 male football players aged 16–33 years, representing the three clubs. The age range was chosen to include the broad range of athletes typically found in amateur football leagues, where players within this age group generally exhibit similar physical capabilities, training levels, and competitive performance. Research suggests that this age range encompasses key stages of athletic development, with the younger group (16–24 years) generally experiencing greater physical development and recovery capacity, while the older group (25–33 years) tends to have more advanced training experience and may face age-related changes in physical function [28,29].

While there are age-related differences in physical capabilities, this range was selected to ensure a comprehensive sample of active football players without introducing excessive variability in athletic performance, as this age range is often seen as a peak period for competitive athletes in football.

Players were selected based on their active participation in club activities, regular training, and competitive matches. Recruitment ensured a diverse sample within the defined demographic range.

The three teams included in this study were selected from Liga4Arad based on their willingness to participate and their geographical accessibility, which facilitated data collection within the resource constraints of the study. These teams were chosen to represent a cross-section of amateur football players; however, it is acknowledged that they may not fully capture the diversity of all 13 teams in the championship. Future research should aim to include a larger and more representative sample of teams to enhance the generalizability of the findings.

The sample size was determined using a power analysis to ensure adequate statistical power for detecting medium to large effect sizes. This calculation was based on the anticipated relationships between nutritional knowledge, dietary habits, physical health, and substance use behaviors. Given the constraints of the population size and study design, the sample size was deemed sufficient for the scope of the study.

Given the small sample size within each age group, presenting the data separately could limit statistical power and reliability. The division into age groups (16–24 years and 25–33 years) was based on existing research suggesting that factors such as training history, physical development, and recovery abilities vary by age, influencing athletic performance and health outcomes. The younger group (16–24 years) generally experiences greater physical development and recovery capacity, while the older group (25–33 years) typically has more advanced training experience and may face age-related changes in physical function. Although the results are presented only as numbers and percentages, the age group division aims to account for these differences and provide deeper insight into how age may impact health and performance.

The exclusion of female athletes from the study is a limitation acknowledged by the researchers. The decision was based on the available male-dominated sample in the specific football league studied. Future research should aim to include female participants to examine potential gender differences in nutritional habits, training behaviors, and performance outcomes.

### 2.3. Inclusion Criteria

The study included football players who met the following criteria:

− Gender: only male football players were included in the study to focus on the specific nutritional and physical health characteristics of this population.
− Age Range: participants aged between 16 and 33 years were eligible to ensure representation of adult football players in their prime athletic years.
− Active Participation: players actively engaged in regular football training sessions at least two to three times a week and actively participated in competitive matches during the study period.
− Willingness to Participate: participants were required to demonstrate a willingness to engage fully in the study, including completing questionnaires and other data collection activities.
− Informed Consent: Written informed consent was a prerequisite for participation. This ensured that players understood the study’s purpose, procedures, potential risks, and the confidentiality measures in place to protect their personal data. For players under 18, parental or guardian consent was also obtained.

### 2.4. Exclusion Criteria

Participants were excluded from the study based on the following criteria:

− Gender: female football players were excluded, as the study aimed to focus solely on the nutritional and physical health characteristics of male athletes.
− Chronic Medical Conditions: individuals with chronic medical conditions such as cardiovascular disease, diabetes, or other illnesses that might affect their nutritional or physical health were excluded to reduce confounding factors.
− Injury or Temporary Health Conditions: players who were currently injured or experiencing temporary health issues that limited their training or match participation were excluded to ensure reliable data.
− Declined Consent: Participants who chose not to provide written informed consent were excluded to uphold ethical standards and ensure voluntary participation. For players under the age of 18, if parental or guardian consent was not obtained, they were also excluded from the study.

### 2.5. Data Collection

Data for this study were collected using a structured questionnaire designed to comprehensively assess the key research objectives. The questionnaire was divided into five distinct sections, each targeting a specific aspect of the participants’ profiles. These sections were carefully constructed to gather both demographic and behavioral data, as well as information about physical health parameters relevant to the study. The sections included the following:

− Section 1: Demographic Information—This section aimed to collect basic participant information, including age, gender, and level of involvement in football activities. Demographic data are crucial for understanding the characteristics of the study sample and identifying potential confounding factors. Age and gender, in particular, are important for assessing how these variables may influence the health and performance outcomes being studied.
− Section 2: Eating Habits—This section focused on participants’ eating patterns, food choices, and adherence to a balanced diet. Eating habits are a key factor in athletic performance and overall health, and this section was designed to capture detailed information about the participants’ dietary choices. The responses helped to assess whether the players are following recommended dietary guidelines and to identify any nutritional gaps that might impact their training or health outcomes.
− Section 3: Nutritional Knowledge—Participants’ understanding of essential nutrition concepts, including macronutrients, vitamins, and minerals, was assessed in this section. Nutritional knowledge is critical for athletes, as it can influence their dietary choices and overall health. By measuring the players’ level of nutritional literacy, this section helped determine if a lack of knowledge might contribute to poor dietary practices that could affect performance or recovery.
− Section 4: Supplements and Substance Use—This section focused on participants’ behaviors related to dietary supplement use, alcohol consumption, and smoking habits. Substance use can significantly impact athletic performance and recovery, so this section was essential for understanding how these factors might affect the players’ health. It also provided insights into whether players are using supplements responsibly and how lifestyle choices may influence their well-being and sports performance.
− Section 5: Sports Activity Levels—This section measured sports activity levels, including training frequency and estimated body fat percentage. Physical activity is a key determinant of athletic performance, and this section was designed to assess how much time players dedicate to training and competition. Estimating body fat percentage also provides important data about physical composition, which can help in understanding performance potential and identifying any physical health issues that may need to be addressed.

The rationale for data collection in this study was to provide a holistic view of the football players’ health and behaviors through a structured questionnaire that directly aligns with the study’s objectives. The questionnaire included five sections, each designed to assess key factors influencing the players’ performance, health outcomes, and overall well-being. By gathering demographic information alongside specific details about lifestyle behaviors, the study aimed to identify potential correlations between eating habits, nutritional knowledge, substance use, and physical activity levels. These factors are essential for understanding and improving athletic health and performance, offering insights that can be used to optimize players’ health strategies and enhance their performance.

#### 2.5.1. Scoring System

The cut-off points for classifying participants’ eating habits and nutritional knowledge into low, moderate, and high categories were established using a self-developed questionnaire with a scoring system. These categories were defined based on predetermined score ranges, which were created for the purposes of this study to ensure meaningful distinctions in the participants’ knowledge and habits. The specific thresholds were determined through an analysis of the score distribution within the sample, reflecting the participants’ varying levels of nutritional knowledge and eating habits.

Each section was scored to provide an overall assessment of participants’ health and lifestyle behaviors:

1. Healthy Eating Habits (Section 2)
  − Low (0–8): poor eating habits; needs significant improvement.
  − Moderate (9–16): some healthy eating practices, but noticeable gaps.
  − High (17–24): consistently healthy eating behaviors.
2. Nutritional Knowledge (Section 3)
  − Low (0–2): limited understanding of nutrition principles.
  − Moderate (3–4): basic knowledge; shows potential for growth.
  − High (5): strong grasp of nutritional concepts and their applications.
3. Supplements and Substances (Section 4)
  − Low Score (0): frequent use of substances; higher risk to health and performance.
  − Moderate Score (1–2): some use of substances; moderate risk.
  − High Score (3): no risky behaviors; excellent discipline.
4. Sports Activity Levels (Section 5)
  − Low (0–3): infrequent or minimal training and match participation.
  − Moderate (4–6): regular training and occasional match participation.
  − High (7–8): intensive training and frequent match participation.

#### 2.5.2. Validation and Data Collection Protocols

The pilot questionnaire was self-developed to explore dimensions specific to the research objectives. It was then refined and validated through a process to ensure its relevance, reliability, and accuracy. The validation process occurred during the initial phase of the study, before the main data collection began, ensuring the questionnaire was both relevant and reliable for the target population. The validation process involved the following steps:

− The General Nutrition Knowledge Questionnaire (GNKQ) was used for assessing nutritional knowledge. While the GNKQ is internationally validated, it has not been specifically validated in Romanian. Therefore, the questionnaire was translated into Romanian and culturally adapted based on the context of the target population. Additionally, the International Physical Activity Questionnaire (IPAQ) was used for assessing physical activity. While the IPAQ Short Form is validated for Romanian populations, it was not entirely appropriate for this study, as it does not fully capture the sports-specific physical activity levels of amateur football players. A more tailored categorization based on frequency and intensity of training and match participation was therefore employed.
− Reliability Testing: the reliability of the questionnaire was assessed using Guttman’s λ2, confirming internal consistency across all sections.

These adjustments helped enhance the accuracy of the data collected, ensuring that the questionnaire effectively addressed the key research dimensions.

Anthropometric measurements, including body weight, height, and body mass index (BMI), were conducted following standard protocols to ensure accuracy and consistency. Specifically, body weight was measured using a digital scale (Omron, model HN-286, Omron Corporation, Kyoto, Japan), calibrated to ensure accuracy to the nearest 0.1 kg. Height was measured using a stadiometer (Seca, model 213, Seca GmbH & Co. KG, Hamburg, Germany), calibrated to ensure accuracy to the nearest 0.1 cm, with participants standing barefoot, ensuring their heels, buttocks, and shoulders touched the wall. BMI was calculated using the following formula: BMI = weight (kg) / height (m^2^). Each measurement was taken twice to ensure consistency, with a third measurement taken if the first two measurements varied by more than 0.5 kg for weight or 1 cm for height. Participants were instructed to wear light clothing and remove shoes for accurate measurement. All measurements were conducted in a quiet and controlled environment to minimize external factors that could influence accuracy.

Data collection adhered to strict ethical guidelines, including participant confidentiality and informed consent, and was conducted in accordance with approved ethical protocols. For participants under the age of 18, parental or guardian consent was obtained prior to participation.

### 2.6. Statistical Analysis

Statistical analyses were performed using JASP 0.19.1 (University of Amsterdam, Amsterdam, The Netherlands). Descriptive statistics were calculated to summarize the data, including means, standard deviations, and percentages. Spearman’s rank correlation coefficients were used to examine relationships between variables such as nutritional knowledge, substance use, and physical parameters. Statistical significance was set at *p* < 0.05.

To assess the reliability of the measurement tools, Guttman’s λ2 was calculated along with its 95% confidence intervals (CI). This analysis confirmed the internal consistency of the data.

The normality of distribution for the relevant variables was assessed using the Shapiro–Wilk test. The results indicated that the data for the variables were not normally distributed (*p*-values < 0.001). Given the non-normal distribution of the data, Spearman’s rank correlation was used to assess the relationships between variables, as it is a non-parametric test that is more appropriate for non-normally distributed data.

### 2.7. Ethical Considerations

The study was approved by the ethical review boards of the participating football clubs in Arad, Romania, prior to commencement:

1. Vointa Macea: protocol code 121/9 September 2023.
2. Soimii Simand: protocol code 103/9 September 2023.
3. Athletico Vinga: protocol code 97/10 September 2023.

Written informed consent was obtained from all participants after explaining the study’s purpose and procedures. For participants under the age of 18, parental or guardian consent was obtained prior to participation. To ensure anonymity while obtaining written informed consent, participants’ identifying information was removed from the data before analysis. While written informed consent was obtained from all participants, their names were not linked to the data collected. A unique participant ID number was assigned to each participant, and all data were coded to protect their identity. This procedure ensured that confidentiality was maintained throughout the study, in line with ethical guidelines. The study adhered to the principles of the Declaration of Helsinki regarding research involving human subjects.

### 2.8. Hypotheses of the Study

1. There is a positive correlation between nutritional knowledge and healthy eating habits among football players.
2. Football players who exhibit healthier eating habits will have a lower body fat percentage.
3. There is a significant negative correlation between the frequency of intense football training sessions and body fat percentage among football players.
4. Higher consumption of dietary supplements among football players is associated with higher alcohol consumption and smoking habits.
5. Football players with greater nutritional knowledge are less likely to engage in unhealthy behaviors such as alcohol consumption and smoking.
6. Football players with higher physical activity levels (measured by training frequency) will have better physical health, as evidenced by lower body fat percentage and improved fitness levels.
7. Football players with higher body fat percentages are more likely to have lower scores in physical fitness measures and participate in fewer intense training sessions.

## 3. Results

This section presents the findings from the study on football players’ nutritional knowledge, dietary habits, substance use, physical parameters, and health behaviors. The data were analyzed to identify patterns in players’ understanding of nutrition, their dietary choices, and their engagement in physical activities and substance use. The results are organized into key areas: nutritional knowledge, dietary habits and choices, supplements and substance use, physical parameters (BMI, body fat, training frequency), and measurement reliability. Statistical analyses, including Spearman’s rank correlations and reliability estimates, were used to assess the relationships between these variables and evaluate the consistency and reliability of the measurements.

### 3.1. Section 1: Demographic Information

The study participants’ demographic information included age and gender. Players were between the ages of 16 and 33 years, and all participants were male. Further demographic data, such as place of residence and education, were not collected as they were not directly relevant to the research objectives of the study.

Table 1 presents the distribution of participants across two age groups, providing both the frequency and percentage for each group:

− 16–24 years: This age group includes 34 participants, representing 56.67% of the total sample. This indicates that the majority of the participants are younger individuals.
− 25–33 years: This age group consists of 26 participants, making up 43.33% of the total sample. While fewer participants fall into this category, it still accounts for a substantial portion of the group.

The results of the multinomial test (Table 2) and the observed vs. expected percentages (Table 3) provide insights into the distribution of variables related to education, socioeconomic status, and physical activity among the study participants.

1. Education:
  − The multinomial test indicates a highly significant association (χ^2^ = 59.333, *p* < 0.001) between observed and expected education levels, implying that the education distribution significantly differs from expectations.
  − Table 3 reveals that the observed percentage of players with primary school education (3.3%) is much lower than the expected 25.0% (CI: 0.4% to 11.5%). In contrast, the observed percentage of players with high school education (65.0%) is significantly higher than expected (25.0%) (CI: 51.6% to 76.9%). The amount of players with a university education (26.7%) aligns closely with the expected value (25.0%) (CI: 16.1% to 39.7%), and postgraduate education (5.0%) is underrepresented compared to the expected 25.0% (CI: 1.0% to 13.9%).
2. Socioeconomic status
  − The multinomial test shows a significant association (χ^2^ = 10.300, *p* = 0.006), indicating that the distribution of socioeconomic status differs significantly from what would be expected by chance.
  − Table 3 shows that the observed percentage of players in the “Low” socioeconomic status category (15.0%) is much lower than the expected value of 33.3%, with a 95% CI ranging from 7.1% to 26.6%. Conversely, a significantly higher proportion of players (48.3%) fall into the “Middle” socioeconomic status group compared to the expected 33.3% (CI: 35.2% to 61.6%). The “High” socioeconomic status category (36.7%) is close to the expected value (33.3%), showing minimal deviation (CI: 24.6% to 50.1%).
3. Physical activity:
  − The multinomial test shows a highly significant difference in physical activity levels (χ^2^ = 27.900, *p* < 0.001), indicating that the distribution of physical activity among the players significantly deviates from the expected values.
  − Table 3 shows that the observed percentage of players in the active but without intense training category (AR) (3.3%) is much lower than expected (33.3%) (CI: 0.4% to 11.5%). In contrast, a significantly higher proportion of players (58.3%) report being in the active with intense football training category (IT) compared to the expected 33.3% (CI: 44.9% to 70.9%). Finally, the observed percentage of players in the very active (VA) category (38.4%) is close to the expected value (33.3%) (CI: 26.1% to 51.8%).

**Table 2:** Results of the multinomial test for education, socioeconomic status, and physical activity (n = 60).

Variable	χ^2^	df	*p*
Education	59.333	3	<0.001
Socioeconomic status	10.300	2	0.006
Physical activity	27.900	2	<0.001

**Table 3:** Observed and expected percentages for socioeconomic status, education, and physical activity (n = 60).

Variable	Level	Observed	Expected: Multinomial	95% Confidence Interval	
				Lower	Upper
Education	Primary School	3.3%	25.0%	0.4%	11.5%
	High School	65.0%	25.0%	51.6%	76.9%
	University	26.7%	25.0%	16.1%	39.7%
	Postgraduate	5.0%	25.0%	1.0%	13.9%
Socioeconomic status	Low	15.0%	33.3%	7.1%	26.6%
	Middle	48.3%	33.3%	35.2%	61.6%
	High	36.7%	33.3%	24.6%	50.1%
Physical activity	AR	3.3%	33.3%	0.4%	11.5%
	IT	58.3%	33.3%	44.9%	70.9%
	VA	38.4%	33.3%	26.1%	51.8%

### 3.2. Section 2: Eating Habits

The participants’ eating habits were assessed using a section of a self-developed questionnaire specifically designed for this study. The questionnaire included several items that covered key dietary behaviors, such as the number of main meals consumed per day, frequency of snacking, consumption of processed foods, sugar and trans-fat intake, and general dietary choices. Each item was rated on a scale with response options designed to capture the frequency or level of each behavior. Table 4 shows the distribution of responses to various dietary habits and practices among the participants.

− Meal Frequency: The majority of participants (53.3%) reported consuming three main meals per day, aligning with traditional meal patterns. A smaller proportion (26.7%) ate four main meals daily, which may indicate a preference for smaller, frequent meals. Notably, 11.7% had only two main meals, and 8.3% limited themselves to a single meal, potentially reflecting intermittent fasting practices or restricted eating routines.
− Snack Consumption: Snacking between meals was moderately common, with 45% consuming snacks “sometimes” and 30% doing so “very rarely”. Frequent snackers accounted for 18.3%, while 6.7% consumed snacks daily.
− Processed Food Consumption: The consumption of processed foods, such as chips and fast food, showed a diverse pattern. While 26.7% reported avoiding processed foods entirely, 41.6% consumed them once a week. Another 26.7% indulged 2–3 times weekly, and 5% ate them daily.
− Sugar and Trans Fat Consumption: When it came to sugar and trans-fat intake, 45% described their consumption as “low”, and 21.7% as “very low”, reflecting a significant awareness of the health risks associated with these substances. Conversely, 28.3% had a “moderate” level of intake, and 5% admitted to “high” consumption.
− Dietary Choices: Dietary decision-making revealed a strong inclination towards planned and health-conscious eating. Approximately 38.3% followed personalized diets tailored for sports performance, and 33.3% adhered to strict and healthy diets. Meanwhile, 15% adopted a balanced approach, attempting to eat healthily while allowing occasional indulgences. However, 13.4% took a less structured approach, eating whatever they liked with little thought to nutrition.

These findings highlight the varied dietary habits and practices among the participants, with most adhering to some form of structured or healthy eating plan while still engaging in some less healthy behaviors, such as occasional processed food or sugar consumption.

Although the questionnaire has not undergone extensive external validation, it was constructed based on established principles of eating behavior in sports nutrition. Future studies will be needed to validate this questionnaire against objective dietary measures.

**Table 4:** Interpretation of dietary habits and choices among football players.

Variable	Level	Frequency	Percent
How many main meals do you eat per day?	4 (4 points)	16	26.7%
	3 (3 points)	32	53.3.7%
	2 (2 points)	7	11.7%
	1 (1 point)	5	8.3%
How often do you consume snacks between meals?	Very rarely (4 points)	18	30%
	Sometimes (3 points)	27	45%
	Frequently (2 points)	11	18.3%
	Daily (1 point)	4	6.7%
How often do you consume processed foods (chips, fast food, etc.)?	Never (4 points)	16	26.7%
	Once a week (3 points)	25	41.6%
	2–3 times a week (2 points)	16	26.7%
	Daily (1 point)	3	5%
How would you describe your consumption of added sugar and trans fats (e.g., sweets, soft drinks)?	Very low (4 points)	13	21.7%
	Low (3 points)	27	45%
	Moderate (2 points)	17	28.3%
	High (1 points)	3	5%
How do you choose your diet?	I follow a personalized diet plan to optimize sports performance (4 points)	23	38.3%
	I follow a strict and healthy diet plan(3 points)	20	33.3%
	I try to eat healthily but indulge occasionally (2 points)	9	15%
	I eat whatever I like without much thought to nutrition (1 point)	5	13.4%
	Missing	0	0.0%
	Total	60	100%

Table 5 illustrates the distribution of knowledge levels about eating habits among football players, based on a questionnaire. The “Eating Habits” are categorized into three levels (low, medium, high), along with their respective frequencies and percentages:

− Low Knowledge: This category includes six participants, representing 10.0% of the total sample. This indicates that only a small fraction of the football players possesses a low level of knowledge about eating habits.
− Medium Knowledge: This group consists of 39 participants, making up 65.0% of the total sample. The majority of the participants fall into this category, highlighting a moderate level of understanding about eating habits.
− High Knowledge: This level includes 15 participants, accounting for 25.0% of the total sample. A notable proportion of players have a high level of knowledge about eating habits, though it is smaller compared to the medium category.

Table 6 presents Spearman’s rank correlation coefficients (*ρ*) and corresponding *p*-values for the relationships between variables related to dietary habits and choices of football players. The variables include meals per day, snack frequency, processed food consumption, added sugar intake, and dietary choices. Below is a detailed interpretation of the results:

− Main Meals and Snacks (*ρ* = 0.675, *p* < 0.001): A strong positive correlation exists between the frequency of main meals (higher scores = more main meals per day) and snack frequency (higher scores = less frequent snacking). This suggests that individuals who consume more main meals per day are less likely to snack between meals, reflecting structured and disciplined eating habits. Regular main meals may help stabilize energy levels, reducing the need for snacking.
− Main Meals and Processed Foods (*ρ* = 0.539, *p* < 0.001): There is a moderate positive correlation between main meal frequency and lower consumption of processed foods (higher scores = less frequent intake). This indicates that individuals who eat more regular meals are less reliant on processed or convenience foods, possibly because they prioritize home-cooked or planned meals over fast-food options.
− Main Meals and Sugar Consumption (*ρ* = 0.540, *p* < 0.001): the moderate positive correlation between main meal frequency and lower sugar consumption (higher scores = less added sugar intake) suggests that individuals who eat structured meals are more likely to maintain a healthier diet overall, limiting sweets and sugary drinks in their routine.
− Main Meals and Diet Choice (*ρ* = 0.563, *p* < 0.001): A moderate correlation shows that eating more frequent main meals is linked to more intentional and healthier diet choices (e.g., personalized plans or strict healthy diets). This highlights the association between meal structure and an overall commitment to health-conscious eating.
− Snacks and Processed Foods (*ρ* = 0.612, *p* < 0.001): A strong positive correlation indicates that individuals who snack less frequently also consume fewer processed foods. This relationship suggests that reducing snacks might limit exposure to processed food items often chosen for convenience.
− Snacks and Sugar Consumption (*ρ* = 0.479, *p* < 0.001): A moderate positive correlation reveals that individuals who snack less frequently also consume lower amounts of added sugar. This underscores the overlap between sugary snacks and overall sugar consumption, emphasizing the health benefits of reducing snacking.
− Snacks and Diet Choice (*ρ* = 0.595, *p* < 0.001): a moderate positive correlation between reduced snacking and healthier diet choices highlights that those who follow structured, health-conscious diets tend to snack less frequently, reflecting disciplined eating patterns.
− Processed Foods and Sugar Consumption (*ρ* = 0.722, *p* < 0.001): The strongest correlation in the data shows a significant relationship between reduced processed food consumption and lower sugar intake. This is expected, as processed foods are often rich in added sugars. Reducing one tends to positively influence the other.
− Processed Foods and Diet Choice (*ρ* = 0.595, *p* < 0.001): A moderate positive correlation suggests that individuals following healthier diet plans are less likely to consume processed foods. Structured dietary choices seem to promote the avoidance of convenience and ultra-processed options.
− Sugar Consumption and Diet Choice (*ρ* = 0.554, *p* < 0.001): A moderate positive correlation between lower sugar consumption and healthier diet choices indicates that structured dietary plans discourage the intake of sweets and sugary beverages. This aligns with the goals of such plans to prioritize nutrient-dense foods over empty calories.

### 3.3. Section 3: Nutritional Knowledge

Table 7 presents the self-reported nutritional knowledge of the football players in the study. The findings reveal varying levels of awareness regarding key nutritional concepts:

− Understanding of BMI: A majority of players (91.7%) reported knowing what BMI means, suggesting a high level of awareness about this basic health metric. Only 8.3% were unfamiliar with BMI.
− Understanding of Fats: When asked about the difference between saturated and unsaturated fats, 63.3% of participants indicated an understanding of the distinction. However, 36.7% admitted a lack of knowledge.
− Knowledge of Vitamins and Minerals: A strong majority (90.0%) reported knowing the primary sources of essential vitamins and minerals, reflecting good awareness of micronutrient sources. However, 13.3% did not have this knowledge.
− Understanding of a Balanced Diet: Most players (86.7%) reported knowing what a balanced diet entails.
− Belief in the Importance of a Balanced Diet for Long-Term Health: A slightly smaller majority (81.7%) believed that a balanced diet influences long-term health. While this is a high percentage, nearly one-fifth of players (18.3%) did not share this belief.

Table 8 illustrates the frequency distribution of nutritional knowledge levels among football players, categorized as low, medium, and high.

− Low Nutritional Knowledge: Only six players (representing 10.0% of the total sample) demonstrated a low level of nutritional knowledge. This suggests that a small proportion of players may lack foundational understanding of essential nutrition concepts.
− Medium Nutritional Knowledge: A total of 25 players (41.7%) exhibited medium levels of nutritional knowledge. This group constitutes a significant portion of the sample, indicating that many players possess some understanding of nutrition.
− High Nutritional Knowledge: The largest group comprises 29 players (48.3%) who demonstrated high levels of nutritional knowledge. This finding indicates that nearly half of the participants are well-informed about nutrition.

This distribution highlights that most players exhibit either high or medium levels of nutritional knowledge, with a smaller proportion falling into the low category.

**Table 8:** Distribution of nutritional knowledge levels among football players.

Age	Frequency	Percent
Low	6	10.0%
Medium	25	41.7%
High	29	48.3%

Table 9 presents the Spearman’s rank correlation coefficients and corresponding *p*-values for associations between various aspects of nutritional knowledge, including awareness of BMI, fats, vitamins and minerals, balanced diet, and long-term health. Below is an interpretation of the results:

− BMI and Other Variables: The correlation between knowledge of BMI and other variables (fats, vitamins and minerals, balanced diet, and long-term health) is non-significant (all *p*-values > 0.05). The association between BMI and knowledge of fats is very weak (*ρ* = 0.021) with a non-significant *p*-value (*p* = 0.874). Similarly, BMI has weak and non-significant correlations with knowledge of vitamins and minerals (*ρ* = −0.101, *p* = 0.445), balanced diet (*ρ* = −0.118, *p* = 0.368), and long-term health (*ρ* = 0.013, *p* = 0.922).
− Fats and Other Variables: Knowledge of fats shows weak and non-significant correlations with other nutritional concepts. The correlation with vitamins and minerals is weak (*ρ* = 0.092, *p* = 0.483). The correlation with balanced diet knowledge is also weak (*ρ* = 0.109, *p* = 0.409). Similarly, the association with long-term health is weak (*ρ* = 0.086, *p* = 0.512).
− Vitamins and Minerals with Balanced Diet and Long-Term Health: The correlation between knowledge of vitamins and minerals and knowledge of a balanced diet is moderately strong and significant (*ρ* = 0.850, *p* < 0.001), suggesting that players who understand vitamins and minerals also understand the concept of a balanced diet. Knowledge of vitamins and minerals also shows a strong and significant correlation with long-term health (*ρ* = 0.704, *p* < 0.001), indicating that those who understand the importance of vitamins and minerals are also more likely to recognize their importance for long-term health.
− Balanced Diet and Long-Term Health: Knowledge of a balanced diet is strongly correlated with long-term health (*ρ* = 0.574, *p* < 0.001), reflecting the belief that understanding a balanced diet directly relates to the awareness of its role in promoting long-term health.

### 3.4. Section 4: Supplements and Substance Use

Table 10 provides an overview of dietary supplement usage, alcohol consumption, and smoking habits among the football players in the study.

− Dietary Supplement Use: The majority of participants (53.3%) reported using dietary supplements for health or performance purposes, indicating a focus on optimizing nutrition or enhancing physical performance. However, 38.3% admitted to using supplements excessively or without professional consultation. A smaller group (8.4%) stated they do not use dietary supplements, instead relying on a well-balanced diet, which reflects adherence to a more natural approach to nutrition.
− Alcohol Consumption: A significant proportion of participants (80%) reported abstaining from alcohol, demonstrating a strong commitment to avoiding this potential health risk. Conversely, 20% admitted to consuming alcohol.
− Smoking: A considerable number of participants (68.3%) reported not smoking, highlighting a majority commitment to avoiding this harmful behavior. However, 31.7% of participants admitted to smoking.

**Table 10:** Substance use and dietary supplementation practices among football players.

Variable	Level	Frequency	Percent
Do you use dietary supplements (e.g., proteins, vitamins, creatine)?	Yes, for health or performance purposes (1 point)	32	53.3%
	No (with a well-balanced diet) (1 point)	5	8.4%
	Yes, excessively or without consultation (0 points)	23	38.3%
Do you consume alcohol?	No (1 point)	48	80.0%
	Yes (0 points)	12	20.0%
Do you smoke?	No (0 points)	41	68.3%
	Yes (1 point)	19	31.7%
	Total	60	100%

Table 11 provides an overview of the distribution of scores in the Supplements and Substances section, which evaluates health risk behaviors among participants.

− Low Score (L): 11 players (representing 18.3% of the sample) exhibited low health risk indicators. This suggests that a minority of participants maintain low-risk health profiles.
− Moderate Score (M): 19 players (31.7%) fall into the medium-risk category. This group comprises a significant portion of the sample, indicating that a notable number of players may have health behaviors or conditions that warrant attention to prevent progression to higher risk levels.
− High Score (H): The largest group, 30 players (50.0%), is categorized as having high health risk indicators. This is a concerning finding, as half of the sample may be at elevated risk for health complications, possibly due to suboptimal dietary behaviors, physical activity levels, or other lifestyle factors.

These findings show that most football players fall into the high- or moderate-risk categories, with only a minority exhibiting frequent risky behaviors.

**Table 11:** Distribution of health risk indicators among football players.

Age	Frequency	Percent
Low	11	18.3%
Medium	19	31.7%
High	30	50.0%

Table 12 presents the Spearman’s rank correlation coefficients (*ρ*) and corresponding *p*-values for the relationships between dietary supplement use, alcohol consumption, and smoking habits among the football players.

− Dietary Supplements and Alcohol Consumption: a moderate positive correlation (*ρ* = 0.548, *p* < 0.001) indicates that higher use of supplements is significantly associated with higher alcohol consumption among participants.
− Dietary Supplements and Smoking: a weak positive correlation (*ρ* = 0.348, *p* = 0.007) suggests that higher use of supplements is also associated with increased smoking behaviors, though the relationship is weaker compared to alcohol.
− Alcohol Consumption and Smoking: a strong positive correlation (*ρ* = 0.734, *p* < 0.001) shows that higher alcohol consumption is significantly associated with higher smoking behaviors.

These results indicate notable interrelationships between the use of supplements, alcohol consumption, and smoking behaviors, with alcohol and smoking showing the strongest association.

**Table 12:** Correlation between dietary supplement use, alcohol consumption, and smoking habits.

Variable		Supplements	Alcohol	Smoking
Supplements	Spearman’s *ρ*	—		
	*p*-value	—		
Alcohol	Spearman’s *ρ*	0.548	—	
	*p*-value	<0.001	—	
Smoking	Spearman’s *ρ*	0.348	0.734	—
	*p*-value	0.007	<0.001	—

### 3.5. Section 5: Sports Activity Levels

Table 13 highlights the BMI distribution among the football players in the study:

− Normal BMI: The majority of players, 41 participants (representing 68.3% of the sample), fall within the normal BMI range. This finding indicates that most football players maintain a healthy body weight.
− Overweight BMI: 19 participants (31.7%) are classified as overweight. Although this group constitutes a smaller portion of the sample, it is still a significant number, suggesting that nearly one-third of the players may face potential challenges in managing their weight.

**Table 13:** BMI distribution among football players.

Age	Frequency	Percent
Normal	41	68.3%
Overweight	19	31.7%

Table 14 presents the estimated body fat percentages and the frequency of intense football training sessions among the players.

− Body Fat Percentage: The estimated body fat percentage among players shows that the majority (53.3%) fall within the 10–20% range, which aligns with typical healthy levels for athletes. Meanwhile, 21.7% of players reported body fat percentages of 21–30%, which, while higher, are still generally manageable. However, 20% of players have body fat levels between 31 and 40%, potentially indicating an elevated risk of decreased performance or health concerns. Additionally, a small proportion (5%) reported body fat percentages exceeding 40%.
− Training Frequency: The training frequency among players demonstrates a strong commitment to physical activity, with 75% participating in four or more intense training sessions per week. This indicates a high level of dedication to their athletic development. Additionally, 25% of players reported training 2–3 times per week, suggesting slightly lower participation in regular intense training compared to their peers. Notably, no players reported training fewer than twice a week.

**Table 14:** Distribution of estimated body fat percentage and training frequency among football players.

Variable	Level	Frequency	Percent
What is your estimated body fat percentage?	10–20% (4 points)	32	53.3%
	21–30% (3 points)	13	21.7%
	31–40% (2 points)	12	20.0%
	40%+ (1 point)	3	5.0%
How often do you participate in intense football training sessions (including matches)?	Never (1 point)	0	0.0%
	Once a week (2 points)	0	0.0%
	2–3 times a week (3 points)	15	25.0%
	4 or more times a week (4 points)	45	75.0%
	Total	60	100%

The correlation in Table 15 analysis reveals a statistically significant positive relationship between estimated body fat percentage and intensive training frequency (Spearman’s *ρ* = 0.341, *p* = 0.008). This indicates that players with higher levels of intensive training tend to have lower body fat percentages, suggesting that regular and intense physical activity is associated with improved body composition among football players in the study group.

Table 16 illustrates the distribution of physical activity levels among participants:

− Low Activity Level: none of the participants reported a low physical activity level (0%), indicating that all football players engage in at least moderate physical activity, which aligns with their participation in the sport.
− Medium Activity Level: 19 participants (31.7%) reported a medium level of physical activity. This group might consist of players with less frequent or less intense training schedules, possibly due to their role in the team, individual training preferences, or other commitments.
− High Activity Level: The majority of participants, 41 players (68.3%), engage in a high level of physical activity. This reflects the demanding training and gameplay schedules typical for football players, emphasizing the physical rigor required in this sport.

### 3.6. Measurement Reliability for Health-Related Items

Table 17 presents the point estimate of Guttman’s λ2 as 0.831, indicating a strong level of internal consistency for the data. The 95% CI for this estimate ranges from 0.799 to 0.875, suggesting that the true value of λ2 is likely to fall within this range with 95% confidence. The λ2 value exceeds the commonly accepted threshold of 0.7, which is generally considered to indicate good reliability. These findings suggest that the measure used in the study is highly reliable, supporting the robustness of the results.

Table 18 presents Guttman’s λ2 reliability estimates for individual items in the dataset, evaluating their contribution to overall reliability. Guttman’s λ2 values are generally high, indicating strong item-level consistency.

− Items such as body fat (λ2 = 0.846) and long-term health (λ2 = 0.839) have the highest reliability values, suggesting they contribute strongly to the overall reliability of the scale.
− Items such as processed foods (λ2 = 0.800) and sugar (λ2 = 0.805) show slightly lower reliability values, indicating a smaller contribution to overall reliability, but they still meet acceptable standards.
− The removal of any single item would not drastically impact the overall reliability, as all λ2 values remain consistently high, demonstrating that each item contributes positively to the scale.

These results confirm the robustness of the scale and the reliability of individual items in assessing nutritional knowledge, health behaviors, and related factors.

**Table 18:** Frequentist individual item reliability statistics (Guttman’s λ2).

Frequentist Individual Item Reliability Statistics	
	If Item Dropped
Item	Guttman’s λ2
Meals/day	0.815
Snacks	0.811
Processed foods	0.800
Sugar	0.805
Diet choice	0.814
BMI	0.837
Fats	0.839
Vitamins/Minerals	0.837
Balanced diet	0.838
Long-term health	0.839
Supplements	0.831
Alcohol	0.830
Smoking	0.834
Body fat	0.846
Intensive training	0.831

## 4. Discussion

The findings of this study provide valuable insights into the nutritional knowledge, dietary behaviors, physical health, and substance use patterns among football players. The analysis was framed around key research questions, with results discussed in the context of the existing literature.

### 4.1. Nutritional Knowledge and Healthy Eating Habits

A significant positive correlation was observed between nutritional knowledge and healthy eating habits, supporting the idea that athletes with a better understanding of nutrition tend to make healthier dietary choices. This aligns with previous studies, which suggest that greater nutritional knowledge helps athletes optimize their dietary patterns to support energy needs, recovery, and overall performance [30,31,32]. This finding underscores the importance of nutritional education in helping athletes make informed choices that can enhance both performance and well-being.

### 4.2. Healthy Eating Habits and Body Fat Percentage

While no strong, direct relationship between healthy eating habits and body fat percentage was found, there was a trend suggesting that healthier eating habits may be associated with lower body fat percentages. This observation is consistent with previous research indicating that a balanced diet can improve body composition in athletes [33,34,35]. However, it is important to recognize that body fat percentage is influenced by multiple factors, including genetics and training intensity. Thus, while nutrition plays a significant role, the relationship between eating habits and body fat may be more complex.

### 4.3. Intense Training and Body Fat Percentage

The data revealed a significant negative correlation between the frequency of intense training sessions and body fat percentage, suggesting that football players who engage in more frequent and intense training tend to have lower body fat percentages. This finding supports the literature, which links high training frequency and intensity with improved body composition and fat reduction [36,37,38]. Intense physical activity enhances muscle mass, boosts metabolism, and promotes fat oxidation, all of which contribute to lower body fat levels [39,40].

### 4.4. Dietary Supplements, Alcohol Consumption, and Smoking

The results partially supported the hypothesis that higher use of dietary supplements correlates with higher alcohol consumption and smoking. A moderate positive correlation was found between supplement use and alcohol consumption, which is consistent with studies identifying a relationship between the use of performance-enhancing supplements and other health-risk behaviors, including alcohol consumption [41,42,43]. However, the correlation with smoking was weaker, indicating that although both behaviors may reflect certain health-risk attitudes, they are likely influenced by different factors. Psychological and social influences warrant further exploration to better understand the complex relationship between supplement use and substance consumption.

### 4.5. Nutritional Knowledge and Unhealthy Behaviors

While the trend suggested that players with better nutritional knowledge were somewhat less likely to smoke or consume alcohol, the results did not show a strong inverse relationship between nutritional knowledge and substance use. This finding aligns with prior research that associates good nutritional knowledge with healthier lifestyle choices in athletes [20,44,45]. However, the influence of psychosocial factors, such as stress, peer pressure, and social norms, should be considered, as these factors can significantly impact substance use behaviors, sometimes outweighing the effects of nutritional knowledge [46,47,48].

### 4.6. Physical Activity and Physical Health

The study confirmed that players with higher levels of physical activity, particularly those who engage in frequent and intense training, exhibit better physical health. These players demonstrated healthier physical parameters, such as lower body fat percentages. This finding reinforces the well-established body of research showing that regular physical activity, especially high-intensity training, is critical for maintaining a healthy body composition and overall physical fitness [49,50,51]. Such training helps maintain optimal health by improving cardiovascular function, muscle mass, and metabolic rate [52]. It is important to note that in this study, the physical activity levels of the amateur football players were categorized into high, moderate, or low levels based on their engagement in football-specific training and match play, rather than general physical activity. This approach was chosen to better reflect the athletes’ actual physical activity levels in the context of their involvement in football, as opposed to general physical activity questionnaires like the IPAQ.

### 4.7. Body Fat Percentage and Physical Fitness

Players with higher body fat percentages tended to score lower on measures of physical fitness, such as training frequency and physical performance. This finding supports the view that lower body fat percentages are typically associated with better athletic performance, including greater endurance and more efficient energy utilization [53,54]. High body fat levels can impede athletic performance by limiting cardiovascular function and muscle mass, thus reducing the ability to perform well during high-intensity training [55,56].

### 4.8. Advantages of the Self-Developed Questionnaire

A notable strength of this study was the use of a self-developed pilot questionnaire, which was tailored to the specific needs of the study population. This allowed for a detailed exploration of the participants’ nutritional knowledge, eating habits, substance use, and physical parameters. The questionnaire was refined during the study, which helped capture a wider range of factors influencing health behaviors. The use of validated instruments in conjunction with the self-developed questionnaire strengthened the reliability and validity of the findings, ensuring that the results were both relevant and comparable to existing research.

The scoring system employed in the questionnaire also allowed for a clear and quantifiable evaluation of participants’ health behaviors, making it easier to categorize and interpret the results. This comprehensive and systematic approach to data collection ensured a thorough analysis of the factors influencing the football players’ health and performance.

### 4.9. Limitations of the Study

This study has several limitations that should be acknowledged. First, the cross-sectional design restricts the ability to establish causal relationships between variables. While the associations observed provide valuable insights, longitudinal studies are needed to examine how nutritional knowledge, training habits and health outcomes evolve over time. This would allow for a better understanding of the directionality and long-term effects of these variables on athletic performance and overall well-being.

Second, the study relied on self-reported data, particularly for dietary habits and substance use, which introduces potential biases such as social desirability and recall inaccuracies. Athletes may overestimate healthy behaviors or underreport harmful habits. To mitigate these biases in future research, objective measures—such as food diaries, nutritional tracking apps, or biomarkers—could provide more accurate and reliable data on participants’ health behaviors.

A key limitation of the study is the relatively small sample size (n = 60), which may limit the generalizability of the findings to a larger or more diverse population of football players. The sample was drawn from a specific league, which may not fully represent all levels or regions of football players. Future research with larger and more diverse samples is recommended to validate the results and ensure broader applicability. Moreover, the sampling design, while adequate, does not fully capture the diversity of football players across different geographic regions, age groups, or competitive levels. The three teams included in this study provided valuable insights into the nutritional knowledge and health behaviors of amateur football players, but they do not represent all teams in Liga4Arad. Expanding the sample to include more teams or different leagues would enhance the generalizability of the findings.

Another limitation is the lack of additional demographic data such as place of residence, education level, or socio-economic background. These factors could provide a more comprehensive understanding of the sample and may influence the interpretation and generalizability of the findings. Future research should consider including a broader range of demographic variables to enhance the context and applicability of the results.

Finally, while this study focused on nutritional knowledge and health behaviors, it did not explore the psychological, social, or environmental factors—such as stress, peer pressure, or team dynamics—that could significantly influence lifestyle choices. These external factors may shape players’ substance use, eating habits, and training habits, and warrant further investigation to better understand the complex determinants of athletes’ health behaviors. The exclusion of female athletes is another limitation of the study. Future research should include female participants to explore potential gender differences in nutritional habits, training behaviors, and performance outcomes.

Additionally, a limitation of this study is the lack of extensive validation of the eating habits questionnaire. While the tool has demonstrated good internal consistency, further research is necessary to establish its construct validity through comparison with objective dietary assessments or other validated questionnaires. Future studies could also assess the stability of the questionnaire over time through test–retest reliability.

### 4.10. Future Directions

To build upon the findings of this study, longitudinal research is needed to examine causal relationships between nutritional knowledge, training habits, and health outcomes. Such studies would provide deeper insights into how these variables interact over time and affect athletes’ performance and well-being in the long term. Future research should focus on tracking the progress of athletes over multiple seasons to understand how changes in nutrition and training impact physical health and performance outcomes.

Furthermore, incorporating objective measures—such as food diaries, nutritional tracking apps, biomarkers, and direct assessments of substance use (e.g., alcohol or nicotine levels)—would help reduce the biases inherent in self-reported data. This approach would ensure more accurate measurement of dietary intake, substance use, and body composition, leading to more reliable findings.

Future studies should also explore psychological and social influences on athletes’ health behaviors. Factors such as stress, social support, and team culture likely play a critical role in shaping players’ choices regarding nutrition, substance use, and physical activity. A deeper understanding of these factors could lead to the development of more targeted and effective interventions aimed at improving athletes’ overall health and performance.

Finally, expanding the sample size to include participants from diverse geographic regions, competitive levels, and age groups would improve the generalizability of the findings. A more representative sample would enable comparisons across different player populations, providing a clearer picture of the factors influencing nutritional and health behaviors in various contexts. This could help tailor interventions to meet the needs of specific groups of athletes, enhancing their health and performance outcomes.

## 5. Conclusions

This study provides valuable insights into the role of nutritional knowledge, dietary behaviors, physical health, and substance use among football players. The findings demonstrate a significant relationship between higher levels of nutritional knowledge and healthier eating habits. Players with a better understanding of nutrition were more likely to consume balanced diets, which in turn positively influenced their physical health and performance. Based on this, it is recommended that football clubs implement targeted nutrition education programs to enhance players’ understanding of healthy eating, which could lead to better dietary choices and improved athletic outcomes.

Additionally, the study highlights the significant role of intense training in reducing body fat percentage and enhancing overall physical fitness. The results show that players engaging in more intense training sessions exhibited better physical health markers. Given this, it is recommended that football teams consider optimizing training schedules to ensure players are getting sufficient intensity in their workouts to maximize body composition and performance benefits.

The self-developed questionnaire used in this study proved to be an effective tool for capturing relevant data on the players’ health behaviors and physical parameters. By tailoring the questionnaire to the specific needs of the football players, the study was able to explore the factors influencing their lifestyle choices in greater depth. Based on these findings, it is recommended that future studies adopt similar, sport-specific tools to better capture the health behaviors and physical attributes of athletes in other sports contexts.

Overall, the results of this study underline the complex relationship between nutrition, physical activity, and substance use. The results suggest that a multifaceted approach is essential for promoting athlete health and well-being. Specifically, comprehensive nutrition education and structured training routines should be prioritized to optimize health and performance. Moreover, targeted interventions focusing on substance use, such as reducing alcohol consumption and promoting healthy habits, could further improve players’ overall health. Implementing such interventions would support football players in making informed, healthier lifestyle choices, ultimately enhancing both their physical well-being and athletic performance.

## Figures and Tables

**Table 1:** Age distribution of male football players participating in the study (n = 60).

Age	Frequency	Percent
16–24	34	56.7%
25–33	26	43.3%

**Table 5:** Distribution of football players’ knowledge levels about eating habits.

Age	Frequency	Percent
Low	6	10.0%
Medium	39	65.0%
High	15	25.0%

**Table 6:** Spearman’s correlations between dietary habits and choices of football players.

Variable		Meals/Day	Snacks	Processed Foods	Sugar	Diet Choice
Meals/day	Spearman’s *ρ*	—				
	*p*-value	—				
Snacks	Spearman’s *ρ*	0.675	—			
	*p*-value	<0.001	—			
Processed foods	Spearman’s *ρ*	0.539	0.612	—		
	*p*-value	<0.001	<0.001	—		
Sugar	Spearman’s *ρ*	0.540	0.479	0.722	—	
	*p*-value	<0.001	<0.001	<0.001	—	
Diet choice	Spearman’s *ρ*	0.563	0.595	0.595	0.554	—
	*p*-value	<0.001	<0.001	<0.001	<0.001	—

**Table 7:** Nutritional knowledge among football players.

Variable	Level	Frequency	Percent
Do you know what BMI means?	Yes (1 point)	55	91.7%
	No (0 points)	5	8.3%
Do you understand the difference between saturated and unsaturated fats?	Yes (1 point)	38	63.3%
	No (0 points)	22	36.7%
Do you know the primary sources of essential vitamins and minerals for the body?	Yes (1 point)	54	90.0%
	No (0 points)	6	10.0%
Do you know what a balanced diet means?	Yes (1 point)	52	86.7%
	No (0 points)	8	13.3%
Do you believe a balanced diet influences long-term health?	Yes (1 point)	49	81.7%
	No (0 points)	11	18.3%
	Total	60	100%

**Table 9:** Spearman’s correlations between nutritional knowledge variables.

Variable		BMI	Fats	Vitamins Minerals	Balanced Diet	Long-Term Health
BMI	Spearman’s *ρ*	—				
	*p*-value	—				
Fats	Spearman’s *ρ*	0.021	—			
	*p*-value	0.874	—			
Vitamins Minerals	Spearman’s *ρ*	−0.101	0.092	—		
	*p*-value	0.445	0.483	—		
Balanced diet	Spearman’s *ρ*	−0.118	0.109	0.850	—	
	*p*-value	0.368	0.409	<0.001	—	
Long-term health	Spearman’s *ρ*	0.013	0.086	0.704	0.574	—
	*p*-value	0.922	0.512	<0.001	<0.001	—

**Table 15:** Correlation between body fat percentage and intensive training frequency.

Variable		Body Fat	Intensive Training
Body fat	Spearman’s *ρ*	—	
	*p*-value	—	
Intensive training	Spearman’s *ρ*	0.341	—
	*p*-value	0.008	—

**Table 16:** Distribution of physical activity levels among participants.

Age	Frequency	Percent
Low	0	0%
Medium	19	31.7%
High	41	68.33%

**Table 17:** Guttman’s λ2 Reliability Estimate for Health-Related Items.

Estimate	Guttman’s λ2
Point estimate	0.838
95% CI lower bound	0.799
95% CI upper bound	0.875

## Data Availability

The raw data supporting the conclusions of this article will be made available by the authors on request.
